# High density mechanical energy storage with carbon nanothread bundle

**DOI:** 10.1038/s41467-020-15807-7

**Published:** 2020-04-20

**Authors:** Haifei Zhan, Gang Zhang, John M. Bell, Vincent B. C. Tan, Yuantong Gu

**Affiliations:** 1 School of Mechanical, Medical and Process Engineering, Queensland University of Technology (QUT), Brisbane, QLD 4001 Australia; 2 Center for Materials Science, Queensland University of Technology (QLD), Brisbane, QLD 4001 Australia; 3 Institute of High Performance Computing, Agency for Science, Technology and Research, 1 Fusionopolis Way, Singapore, 138632 Singapore; 4 University of Southern Queensland, Springfield Central, QLD 4300 Australia; 5 Deparment of Mechanical Engineering, National University of Singapore, 9 Engineering Drive 1, Singapore, 11576 Singapore

**Keywords:** Energy storage, Mechanical engineering, Nanoscale materials

## Abstract

The excellent mechanical properties of carbon nanofibers bring promise for energy-related applications. Through in silico studies and continuum elasticity theory, here we show that the ultra-thin carbon nanothreads-based bundles exhibit a high mechanical energy storage density. Specifically, the gravimetric energy density is found to decrease with the number of filaments, with torsion and tension as the two dominant contributors. Due to the coupled stresses, the nanothread bundle experiences fracture before reaching the elastic limit of any individual deformation mode. Our results show that nanothread bundles have similar mechanical energy storage capacity compared to (10,10) carbon nanotube bundles, but possess their own advantages. For instance, the structure of the nanothread allows us to realize the full mechanical energy storage potential of its bundle structure through pure tension, with a gravimetric energy density of up to 1.76 MJ kg^−1^, which makes them appealing alternative building blocks for energy storage devices.

## Abstract (web summary)

Carbon nanothreads are promising for applications in mechanical energy storage and energy harvesting. Here the authors use large-scale molecular dynamics simulations and continuum elasticity theory to explore mechanical energy storage in carbon nanothreads-based bundles.

## Introduction

Energy storage is a key bottleneck in the supply of renewable energy resources to the wider economy. Currently, extensive research is in progress, directed towards solving the supply of renewable energy by utilizing industrial waste heat, solar photovoltaic energy and harvesting mechanical energy in the environment. Diverse energy harvesters have been developed, such as electromagnetic electric energy generators, mechanical energy harvesters and electrochemical harvesters. However, the storage of intermittent renewable energy supplies means that large-scale energy storage is becoming an essential component of the twenty-first century energy system. The high strength and high modulus of carbon nanotube (CNT) makes the utilization of CNT-based fibres as a mechanical energy storage medium^1^, and as an energy harvester^2^ viable. Comparing with the electrochemical batteries (e.g. Li-ion batteries)^3^, CNT fibre-based mechanical energy storage medium allows fast efficient energy charging and discharging, and tends to be much more stable and reversible (with a high cycle performance)^4^. These unique features make them promising building blocks for artificial muscles^5^, soft robotics^6^ and flexible electronics^7^.

In general, CNT fibres are comprised of axially aligned and densely packed individual CNTs, which can be fabricated through either a spinning^8^ or twisting/rolling technique^9^. Depending on the applied techniques, different CNT fibre structures have been reported, such as knitted structures^10^, parallel structures or twisted structures^11^. Due to the complexity of their structures and various post treatments (e.g. liquid shrinking, infiltration^12^, functionalization, etc.), there is a large variation in the mechanical performance of CNT fibres^13^. Their strength ranges from 0.23 to 9.0 GPa, and the modulus ranges from 70 to 350 GPa^14^. Very recent work reported the fabrication of CNT bundles with a tensile strength over 80 GPa, based on ultralong defect-free CNTs^15^. With CNTs of large diameters, such as the most abundant (10,10) and (18,0) CNTs, the corresponding CNT bundle/ropes are metastable due to the flattening of the constituent CNTs during deformation^16^. Such flattening phenomenon will adversely affect the mechanical performance of the fibre. As such, extensive efforts have been made to fabricate CNT fibres with good and controllable mechanical performance^17^.

In 2015, a class of one-dimensional (1D) carbon nanostructure—the carbon nanothread—was reported^18^. Although experimental efforts are still ongoing to characterize the atomic structures of carbon nanothreads^19,20^, first-principle calculations have systematically enumerated the potential configuration of carbon nanothreads, including the fully saturated (or degree-6) and partially saturated (or degree-4) structures^21,22^. Unlike the *sp*^2^ bonding in CNT, the carbon nanothread is an ultra-thin *sp*^3^-bonded carbon structure. They offer an exciting opportunity to overcome the limitations of CNT fibres and can be fabricated with consistency into high strength carbon nanothread bundles. It has a fully hydrogenated surface that allows for the introduction of interfacial covalent bonds between carbon nanothreads^23^, while retaining the thread-like morphology and their excellent mechanical properties^24^. Its non-smooth surface can trigger strong mechanical interlocking effect between carbon nanothreads^25^, or with a polymer matrix^26^. Preliminary studies have shown that nanothreads have excellent mechanical properties comparable with those of CNT, for example, a high stiffness of ~850 GPa and a bending rigidity of ~5.35 × 10^−28^ N·m^2^ ^27,28^, and a structural-dependent ductility^29^. The carbon nanothread is also reported to possess a tailorable thermal conductivity^30,31^. Particularly, researchers have already reported the synthesis of single-crystalline packing of nanothreads (across hundreds of microns)^32^. This opens up a facile way to exfoliate nanothread bundles. It is thus of great interest to assess the mechanical energy storage capacity of a nanothread bundle. With a combination of large-scale molecular dynamics (MD) simulations and elasticity theory, this work explores contributions from different deformation modes to the energy storage capacity in nanothread bundles. It is shown that mechanical energy storage of nanothread bundles in a pure tensile mode surpasses that of advanced Li-ion batteries.

## Results

Considering the 1D nature of carbon nanothread, we first compare the energy storage capacity of nanothread bundles with the extensively studied CNT bundles and take the most abundant (10,10) armchair CNT as the benchmark. There are different approaches being developed to describe the mechanical deformation of bundle structures, such as the analytical^33,34^ and the theoretical models^16,35^. The former requires additional well-defined properties of nanothreads under continuum description, such as Poisson’s ratio and bending moment, which are ambiguous for ultra-thin nanothreads. On the other hand, theoretical models can be based on the general Hooke’s law, which bypasses the direct continuum approximation and focuses on the linear elastic deformation regime. This work adopts the theoretical model to assess the energy storage of the nanothread bundle structure following the framework established previously by Tománek and co-authors^16,35^ for CNT. For this purpose, we first acquire the mechanical properties and energy storage capability of an individual nanothread under four different deformation modes that are occurring in a bundle structure under torsion, including torsion, tensile, bending and radial compression, and then assess the mechanical energy storage of a twisted bundle structure. The commonly used metric–gravimetric energy density (also known as specific energy density) was adopted to describe the energy storage capacity.

### Mechanical strain energy in an individual nanothread

**Fig. 1:**
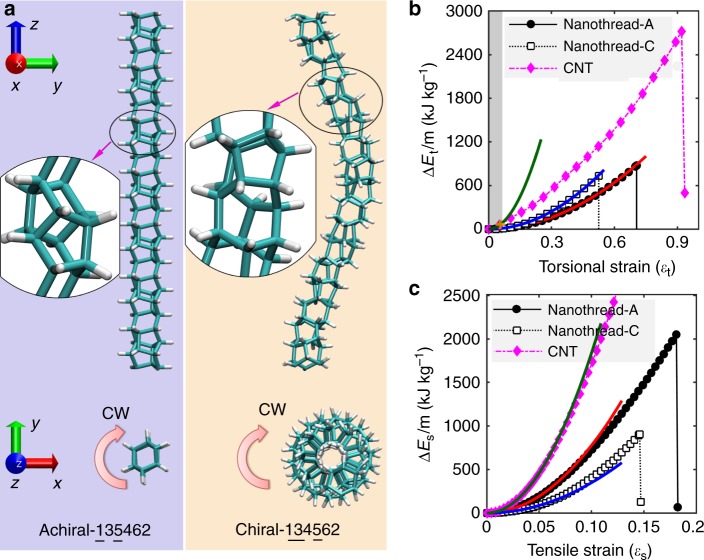
Strain energy density of an individual nanothread and CNT from MD simulations. **a** Atomic configurations of nanothread-A (left, 135462) and nanothread-C (right, 134562). The six integers (some underscored) represent the bonding topology in the structure. Upper panels are side views, and bottom panels are end-on views. CW refers to the clockwise torsion. **b** Torsional strain energy density vs. the dimensionless torsional strain. **c** Tensile strain energy density vs. the tensile strain. Solid lines in **b**, **c** are the corresponding fitting curves based on Hooke’s law.

Figure 1a illustrates the two different nanothreads being considered, which are denoted as nanothread-A and -C, respectively. Nanothread-A has a symmetrical cross-section and linear morphology, while nanothread-C possesses an initial helical morphology. Several studies already reported the mechanical behaviours of individual nanothreads under tensile and bending; here we re-visit these deformation modes for comparison consistency. Figure 1b compares the strain energy density ${\mathrm{\Delta }}E_{\mathrm{t}}/m$ (kJ kg^−1^) as obtained from MD simulation for the two selected nanothreads under torsion. Owing to its small diameter, the nanothread is able to achieve a very high torsional angle before any bond breakage, ~25.55 and 17.28 rad for nanothread-A and -C, respectively. Here, the dimensionless torsional strain is adopted to measure the deformation of the sample, which is calculated from $\varepsilon _{\mathrm{t}} = \varphi D_0/l_0$, with $D_0$ and $l_0$ as the equivalent diameter and length of nanothread and *φ* as the twist angle. The torsional elastic limit is thus defined as the maximum dimensionless torsional strain before the occurrence of irreversible deformation (i.e. fracture or bond breakage), and the gravimetric energy density refers to the strain energy density at the elastic limit. We found that nanothread-A has a slightly higher gravimetric energy density (~884 kJ kg^−1^) than that of nanothread-C (~737 kJ kg^−1^), and the torsional elastic limit $\varepsilon _{\mathrm{t,max}}$ for nanothread-A is ~0.71, which is also higher than that of the nanothread-C (~0.51), that is, nanothread-A can sustain larger torsional deformation. Note that nanothread-C is twisted along the direction of the spiral in Fig. 1b, which is referred as clockwise torsion and being emphasized in this work (see Supplementary Fig. 1 for the anti-clockwise torsion of nanothread-C).

Compared with nanothread, CNT has a large fracture strain of ~0.92, while the corresponding torsional angle is much smaller (only ~10.11 rad). Most importantly, CNT is found to exhibit flattening at a very small torsional angle of only 0.63 rad (i.e. $\varepsilon _{\mathrm{t}}$ ~ 0.06), which does not occur in nanothreads. The gravimetric energy stored is only ~65 kJ kg^−1^ before flattening, more than one order smaller than the nanothread at the elastic limit. We associate the limit of the elastic regime with the onset of flattening. Further inspection of the atomic configuration reveals that CNT undergoes three distinct elastic deformation stages before fracture, including initial elastic deformation, flattening, and torsion without further flattening (see Supplementary Fig. 2). The eventual failure of CNT occurs when $\varepsilon _{\mathrm{t}} \sim 0.92$ with a high gravimetric energy density of 2720 kJ kg^−1^.

Figure 1c compares the tensile deformation of the two nanothreads. Here, the engineering tensile strain is adopted, which is calculated from $\varepsilon _{\mathrm{s}} = (l - l_0)/l_0$, with $l_0$ and *l* as the length of the initial and stretched nanothread, respectively. The elastic limit is ~0.18 and 0.15 for nanothread-A and -C, respectively, which are consistent with that reported in the literature^28,36^. Compared with torsion, the nanothread exhibits a much higher strain energy storage capacity under tensile deformation. The gravimetric energy density is ~2051 and 906 kJ kg^−1^ for nanothread-A and -C, respectively. For CNT(10,10), MD predicts an elastic limit of ~0.22, which is consistent with the results from first-principle calculations^35^. The experimentally measured elastic limit of ~0.12 is much smaller than the predicted value due to the existence of defects^37^. This yields to a strain energy density of ~2374 kJ kg^−1^ in Fig. 1c. From the MD simulation, the maximum energy density is ~6810 kJ kg^−1^ for CNT before bond breakage is observed.

**Fig. 2:**
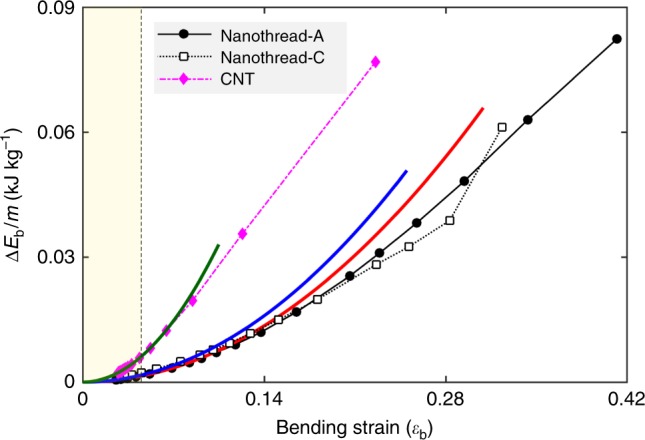
Bending strain energy density as a function of bending strain. Solid lines are the corresponding fitting curves based on Hooke’s law.

Figure 2 shows the bending strain energy density ${\mathrm{\Delta }}E_{\mathrm{b}}/m$ (kJ kg^−1^) as a function of the bending strain. The bending strain is defined as $\varepsilon _{\mathrm{b}} = D_0/R$ (with *R* as the local radius of the bending curvature) and the bending direction is along *y*-axis as shown in Fig. 1a. According to the atomic configurations, the bending elastic limit is ~0.34 and 0.26 for nanothread-A and -C, respectively. These correspond to a low gravimetric energy density of ~468 and 288 kJ kg^−1^, respectively. In line with previous works^38^, CNT experiences apparent bending induced buckling at a very small strain of ~0.05 (see Supplementary Fig. 3 for more details), which corresponds to a bending strain energy density of only ~49.3 kJ kg^−1^. The maximum curvature before any bonds breakage is ~0.23 (with the structure experiencing severe buckling), which yields to a high energy density of 618.6 kJ kg^−1^.

**Fig. 3:**
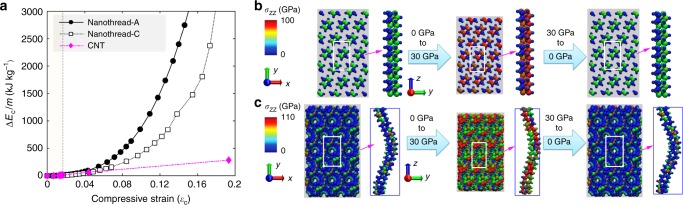
Radial compression of nanothread triangular lattice from simulation. **a** The compressive strain energy density as a function of compressive strain for nanothread-A, nanothread-C, and CNT(10,10). **b** Nanothread-A lattice compressed from 0 GPa (left panel) to 30 GPa (middle panel) and relaxed to 0 GPa (right panel). **c** Nanothread-C lattice compressed from 0 GPa (left panel) to 30 GPa (middle panel) and relaxed to 0 GPa (right panel). Atoms in **b**, **c** are coloured according to the axial virial stress. In each panel, left images show the periodic triangular lattice (in top-view) and right images show a representative individual nanothread (in side-view) in the triangular lattice.

Figure 3 shows that the radial compressive strain energy density ${\mathrm{\Delta }}E_{\mathrm{c}}/m$ (kJ kg^−1^) increases with decreasing separation distance or increasing radial compressive strain. The radial compressive strain in the linear regime of small deformations is estimated from $\varepsilon _{{\mathrm{c}},ij} = 1 - d_{ij}/d_0$. Here, $d_{ij}$ is the nanothread inter-tubular distance under different pressure. Nanothread-A exhibits a very high elastic limit of ~0.19 together with a high gravimetric energy density of ~6051 kJ kg^−1^. In comparison, nanothread-C shows a smaller elastic limit of ~0.18 and a gravimetric energy density of ~3063 kJ kg^−1^. The big difference between these two nanothreads stems from their structural differences. As shown in Fig. 3b, c, nanothread-C with its initial helical morphology experiences a substantial lateral deformation, which leads to earlier bond breakage with the further increment of pressure (see Supplementary Fig. 4 and Supplementary Videos 1 and 2 for more details).

According to the simulation results, CNT experiences structural change or flattening once the radial pressure exceeds ~1.4 GPa. Because of the flattening, the compressive strain of CNT changes suddenly from ~0.017 to 0.04 when the radial pressure increases from 1.4 to 1.5 GPa (Fig. 3a) because of the remarkable change in the inter-tubular distance. The compressive strain before the structural change is only ~0.017, corresponding to an extremely small strain energy density of ~14.1 kJ kg^−1^. The final fracture strain where bond breakage and formation occurs is ~0.33 (with an energy density of 4053 kJ kg^−1^), when all CNTs are completely flattened (see Supplementary Fig. 4). As the CNT experiences flattening during compression, the radial compressive strain relative to the initial inter-tubular distance after flattening is only intended as a placeholder for the strain calculation (as a convenient convention for discussion).

### Mechanical strain energy in carbon nanothread bundles

**Fig. 4:**
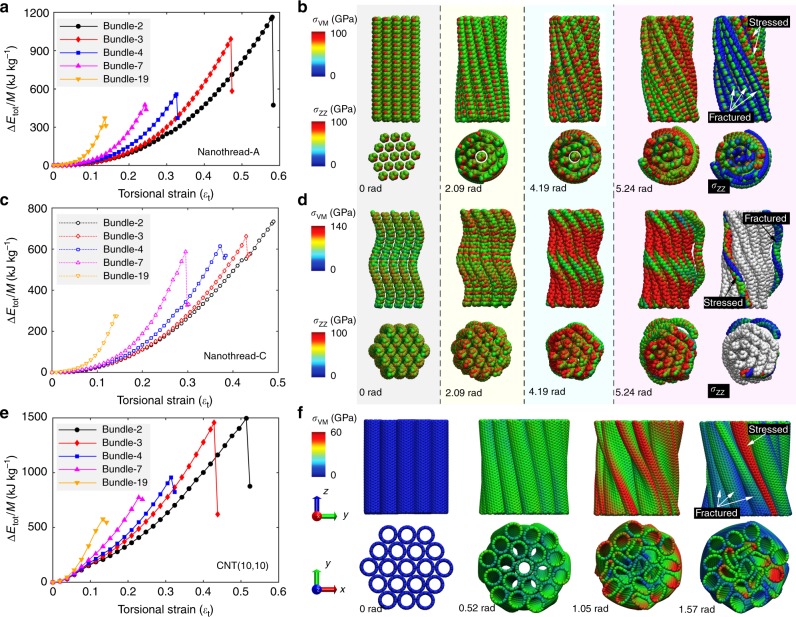
Mechanical energy storage in bundle structures from simulation. **a** Strain energy density as a function of torsional strain of nanothread-A bundles and **b** the corresponding atomic configurations of bundle-19. The last column is coloured based on axial atomic virial stress and the rest is coloured based on the atomic Von Mises (VM) stress; **c** strain energy density as a function of dimensionless torsional strain of nanothread-C bundles; and **d** the corresponding atomic configurations of bundle-19. For clarity, in the last column only one fractured and one connected (stressed) filaments are coloured based on axial atomic virial stress; **e** strain energy density as a function of the dimensionless torsional strain of CNT bundles; and **f** the corresponding atomic configurations of bundle-19. For **b**, **d**, **f**, the atomic configurations show only a middle segment (~6 nm) of the whole sample, and only C atoms are visualized for nanothread bundles.

Following, we consider the mechanical energy stored within twisted nanothread bundles. Different bundles are denoted as bundle*-n* with *n* representing the filament number varying from 2 to 19. Figure 4a, c compare the strain energy density $\Delta E_{\mathrm{tot}}/M$ (kJ kg^−1^) as a function of the dimensionless torsional strain for different nanothread-A and -C bundles (with $\Delta E_{\mathrm{t}}$ and *M* as the total strain energy and the total mass of the bundle, respectively). As expected, nanothread bundles with larger number of filaments possess higher strain energy density at the same torsional strain (within the elastic regime). For each bundle, the strain energy density exhibits a parabolic relationship with the torsional strain. More interestingly, the elastic limit (and the gravimetric energy density) of the nanothread bundles decreases remarkably when the number of filaments increases. The dimensionless torsional strain limit is ~0.47 for nanothread-A bundles with three filaments (with a gravimetric energy density of 991 kJ kg^−1^), which is more than 2.5 times higher than its counterpart with 19 filaments (~370 kJ kg^−1^ with a dimensionless torsional strain limit of ~0.14). Similar results are also observed for nanothread-C bundles, that is, the bundle with three filaments has an elastic dimensionless torsional strain limit of ~0.43; it is only ~0.14 for the counterpart with 19 filaments. Surprisingly, the CNT(10,10) bundles are found to possess similar gravimetric energy density as that of nanothread counterparts, although individual CNT(10,10) has a much higher Young’s modulus. For example, the bundle with 19 filaments has an elastic limit of ~0.13 and a gravimetric energy density of ~577 kJ kg^−1^. Unlike nanothread bundles, the flattening of individual CNT during torsion causes a deviation of the energy density profile from a parabolic relationship with the torsional strain (see Fig. 4c). It is notable that although the elastic limit of CNT bundle in terms of dimensionless torsional strain is comparable with that of nanothreads, the actual torsional angle is very small. For instance, the torsional angle at the elastic limit is ~4.92 and 4.71 rad for bundle-19 constructed from nanothread-A and -C, respectively, whereas the CNT-based bundle can only sustain less than one-third of this torsional deformation (~1.47 rad).

Figure 4b, d, f illustrate the representative deformation processes of nanothread and CNT bundles (also see Supplementary Videos 3–5). For nanothread-A bundles, each initial filament is straight and the deformation of the bundle is similar to that constructed from continuum straight rods^33,39^. It is found that the stress accumulates faster at the outer layer of nanothread bundles during torsion, which experiences the earliest fracture. Further simulations show that the sample length exerts negligible influence on the torsional behaviour of the investigated bundles in this work (see Supplementary Fig. 5, Supplementary Fig. 6 and Supplementary Note 1 for details). It is interesting that the fractured nanothreads (at the outer surface) remain in a curved profile for a certain torsional strain. According to the atomic configurations at the twist angle of 5.24 rad in Fig. 4b (right panel), the axial stress is largely released after fracture. In other words, the curved profile is maintained by the inter-filament van der Waals (vdW) interactions, which further suggests a low bending stiffness of the nanothread-A. In comparison, due to its helical characteristic, the nanothread-C bundles are analogous to second-level hierarchical helical structures. During torsion, each filament will coil further, leading to smaller pitch length and local torsion-induced buckling. Again, outer layer filaments experience higher stress and earlier fracture. From the atomic configuration at the torsional angle of 5.24 rad in Fig. 4d (right panel), the stress status of each nanothread-C filament is analogous to that under tensile deformation, that is, they exhibit a double-helix stress distribution with one helix experiencing tensile and the other experiencing minor compressive stress^28^. In line with the low bending stiffness, nanothread-C is also observed to retain a curved profile after fracture. As expected, the CNT bundle exhibits a similar deformation process, while individual CNTs experience severe flattening during torsion that is not seen in nanothread based bundles.

### Mechanical energy storage under continuum elasticity description

Hooke’s law is adopted to describe the deformation of an individual nanothread under each deformation mode in the linear elastic regime, following the framework established for CNT^16,35^. Generally, the strain energy density is related to the strain through $\Delta E_X/m = k_X\varepsilon _X^2$, where $\Delta E$ is the strain energy; $m$ is the sample mass; $k$ is the elastic constant (in the unit of MJ kg^−1^); $\varepsilon$ is the strain; and $X$ stands for the torsion, stretching, bending, and compression. The constant $k$ that is related to each deformation mode can be obtained from quadratic fitting functions based on the MD results. For the twist deformation (Fig. 1b), the torsional constant ($k_t$) is ~2.70 MJ kg^−1^ for nanothread-C and 1.78 MJ kg^−1^ for nanothread-A. As shown earlier, the elastic deformation of CNT(10,10) can be divided into three stages, which correspond to a torsional constant of ~19.76 MJ kg^−1^ (with $\varepsilon _t \,<\, 0.06$ before the occurrence of flattening), 4.74 MJ kg^−1^, and 2.78 MJ kg^−1^, respectively (see Supplementary Fig. 2).

For tensile deformation (Fig. 1c), the strain energy curve of nanothread-A follows the parabolic relationship initially, and then exhibits a softer response, which is similar to the tensile deformation of an individual CNT^16^. In comparison, nanothread-C shows an enhanced response after the initial harmonic regime, which is caused by its helical morphology^28^. The helical nature of the structure causes the nanothread to undergo a combination of torsion and tension when it is stretched^33^. Based on the quadratic fitting, nanothread-A has a tensile constant ($k_{\mathrm{s}}$) of 77.60 MJ kg^−1^ and nanothread-C has a $k_{\mathrm{s}}$ of 34.81 MJ kg^−1^. In comparison, the CNT(10,10) exhibits a tensile constant of 183.51 MJ kg^−1^. In terms of bending deformation (Fig. 2), bending constants ($k_{\mathrm{b}}$) of 5.12 and 6.04 MJ kg^−1^ are obtained for nanothread-A and -C, respectively. For the CNT(10,10), the MD results are best reproduced by a $k_{\mathrm{b}}$ of 24.11 MJ kg^−1^ before the occurrence of buckling ($\varepsilon _{\mathrm{b}} \,<\,0.05$). From Fig. 3, compression constants ($k_{\mathrm{c}}$) of 43.74 and 31.80 MJ kg^−1^ are derived for nanothread-A and -C, respectively. For the CNT(10,10), the compression constant is ~48.72 MJ kg^−1^ before the occurrence of structural change.

Table 1 lists all mechanical properties extracted and fitted from MD simulation results. It is worth noting that the elastic constants and the gravimetric energy densities of CNT related to torsion, tension and bending are all larger than those of the carbon nanothreads, suggesting that CNT has better energy storage capacity under these deformation scenarios. Based on Hooke’s law, the total strain energy for a twisted bundle with *n* filaments can be quantified from $\Delta E_{\mathrm{tot}} = {\sum} {(\Delta E_{\mathrm{t}} + \Delta E_{\mathrm{s}} + \Delta E_{\mathrm{b}} + \Delta E_{{\mathrm{c}},ij})}$ for small deformation. Considering the total mass of the bundle $M = nm$, the strain energy density ${\mathrm{\Delta }}E_{\mathrm{tot}}/M$ is derived as^16,35^,

1 $$\Delta E_{\mathrm{tot}}/M = \frac{1}{n}\left[ {nk_{\mathrm{t}}\varepsilon _{\mathrm{t}}^2 + k_{\mathrm{s}}\mathop {\sum}\limits_{i = 1}^n {\varepsilon _{{\mathrm{s}},i}^2 + k_{\mathrm{b}}\mathop {\sum}\limits_{i = 1}^n {\varepsilon _{{\mathrm{b}},i}^2 + k_{\mathrm{c}}\mathop {\sum}\limits_{i < j} {\varepsilon _{{\mathrm{c}},ij}^2} } } } \right].$$

Here, $\varepsilon _{\mathrm{t}} = \varphi D_0/l_0$; $\varepsilon _{{\mathrm{s}},i} = \sqrt {1 + \left( {\rho _i\varphi /l_0} \right)^2} - 1$; $\varepsilon _{\mathrm{b}} = D_0/R$, with $R_i = \rho _i[1 + \left( {l_0/\varphi \rho _i} \right)^2]$^40^; $\varepsilon _{{\mathrm{c}},ij} = 1 - d_{ij}/d_0$; $D_0$ is the equivalent diameter of nanothread; and *n* is the filament number. For the torsional deformation within a bundle configuration, each filament has a constant rotational or coil radius $\rho _i$, which is the equilibrium separation distance between the filament axis and the rope axis. For simplicity, we consider each filament has the same equilibrium distance *d*_0_ to its closest neighbours. It is thus evident that the dimensionless torsional strain ($\varepsilon _{\mathrm{t}}$) is the same for each nanothread filament, which equals to the overall torsion strain. The coil radius can be related to the closest neighbour inter-tubular distance ($d_{ij}$) through $\rho _i = d_{ij}/2{\mathrm{sin}}(\pi /n)$ if the filament number *n* is <7. As such, both tensile strain and bending strain can be correlated with the dimensionless torsional strain and compressive strain using Taylor series. Here, we consider the bundles with $n \le 7$ filaments (see Supplementary Fig. 7 and Supplementary Note 2 for more details and bundles with larger *n*), which form a regular polygon cross-section (such as the bundle-3 or bundle-4). In this regard, the tensile strain and bending strain can be re-expressed as $\varepsilon _{{\mathrm{s}},i} = \eta ^2d_0^2\left( {1 - \varepsilon _{\mathrm{c}}} \right)^2\varepsilon _{{\mathrm{t}},i}^2/2D_0^2$ and $\varepsilon _{{\mathrm{b}},i} = \eta d_0( {1 - \varepsilon _{{\mathrm{c}},ij}} )\varepsilon _{{\mathrm{t}},i}^2/D_0$, respectively. Here, $\eta = \left[ {2\sin \left( {\pi /n_1} \right)} \right]^{ - 1}$. Thus, the total strain energy density can be written as a function of compressive strain and torsional strain:

2 $$E_{\mathrm{tot}}\left( {\varepsilon _{\mathrm{t}},\varepsilon _{\mathrm{c}}} \right)/M \approx \, \frac{1}{n}\left[ nk_{\mathrm{t}}\varepsilon _{\mathrm{t}}^2 + n_1\frac{{k_{\mathrm{s}}}}{4}\left( {\frac{{\eta d_0}}{{D_0}}} \right)^4\left( {1 - \varepsilon _{\mathrm{c}}} \right)^4\varepsilon _{\mathrm{t}}^4 \right. \\ \left. +\, n_1k_{\mathrm{b}}\left( {\frac{{\eta d_0}}{{D_0}}} \right)^2\left( {1 - \varepsilon _{\mathrm{c}}} \right)^2\varepsilon _{\mathrm{t}}^4 + n_{\mathrm{c}}k_{\mathrm{c}}\varepsilon _{\mathrm{c}}^2 \right],$$

where $n_1$ is the number of filaments experiencing tension and bending (which equals to $n - 1$ and *n* for the bundle with and without a central filament, respectively), and $n_{\mathrm{c}}$ is the number of pair filaments. For a given torsional strain $\varepsilon _{\mathrm{t}}$, the compressive strain $\varepsilon _{\mathrm{c}}$ can be calculated by considering the system with a minimum strain energy, that is, $\partial E_{\mathrm{tot}}/\partial \varepsilon _{\mathrm{c}} = 0$.

**Table 1:** Mechanical properties of carbon nanothreads and CNT(10,10) as derived from MD simulations.

Deformation mode	Type	Elastic constant $k$ (MJ kg^−1^)	Elastic limit $\varepsilon _{\mathrm{max}}$	Gravimetric energy density ∆*E*/*m* at $\varepsilon _{\mathrm{max}}$ (kJ kg^−1^)
Torsion	A	1.78	0.71	884
	C	2.70	0.51	737
	CNT	19.76 (4.74^a^, 2.78^b^)	0.06 (0.92)	65 (2720)
Tension	A	77.60	0.18	2051
	C	34.81	0.15	906
	CNT	183.51	0.12 (0.22)	2374 (6810)
Bending	A	5.12	0.34	468
	C	6.04	0.28	288
	CNT	24.11	0.05 (0.23)	49.3 (618.6)
Compression	A	43.74	0.19	6051
	C	31.80	0.18	3063
	CNT	48.72	0.017 (0.33)	14.1 (4053)

A and C represent nanothread-A and nanothread-C, respectively.

^a,b^The torsional elastic constants for CNT at the second and third elastic deformation stages. For CNT, the values within the parentheses refer to the elastic limit and the corresponding gravimetric energy density before the onset of fracture, and the values outside the brackets refer to the elastic limit and the corresponding gravimetric energy density before structural change, that is, flattening or buckling.

**Fig. 5:**
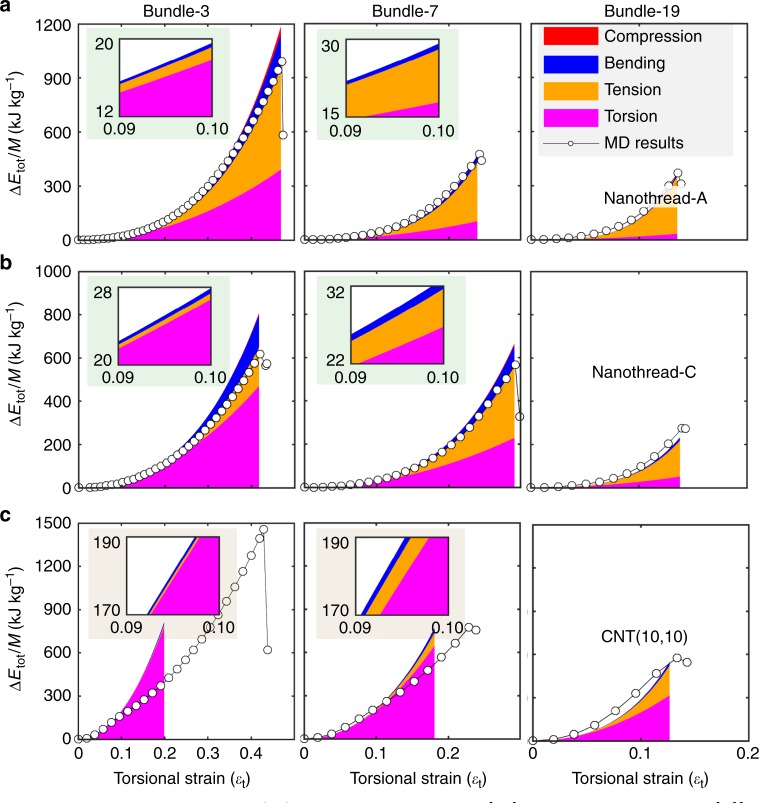
Theoretical predictions of the contributions of different deformation modes. Strain energy density for bundle-3, -7 and -19 that are comprised of by: **a** Nanothread-A; **b** Nanothread-C and **c** CNT(10,10).

Figure 5 compares the theoretically predicted strain energy components for the nanothread bundle-3, -7 and -19 at different torsional strains. Focusing on the low strain region (e.g. with $\varepsilon _{\mathrm{t}}\, <\,10\%$), good agreement is observed between the predicted strain energy and MD simulation results. This demonstrates the capability of the theoretical model to quantitatively describe the strain energy storage and to distinguish the contributions from different deformation modes in the linear elastic region. From Fig. 5, torsion and tension are the two dominant modes for the mechanical energy storage for both nanothread-A and -C bundles. Specifically, torsion dominates energy storage at low strain for bundles with a small filament number, and the tension becomes dominant when the filament number increases (as illustrated in insets of Fig. 5a, b). Such observation is reasonable as the tensile strain increases faster than torsional strain for bundles with large filament number due to the increased bundle radius. For all bundles examined, the compression exhibits a negligible contribution to the total deformation energy, followed by the bending deformation.

Despite the fact that the theoretical model (Eq. 2) is established for the linear elastic regime with small deformation, we find that the predicted energy density curves are in good agreement with those obtained from MD simulations when $\varepsilon _{\mathrm{t}}$ is extended to the elastic limit (Table 1). For instance, the predicted maximum gravimetric energy density is ~1190, 471 and 366 kJ kg^−1^ for nanothread-A bundles with 3, 7 and 19 filaments, respectively, which are very close to those obtained in the simulation (~991, 474 and 370 kJ kg^−1^, respectively). Similar results are found for nanothread-C based bundles. This consistent result suggests the applicability and effectiveness of the theoretical model for a qualitative analysis of the strain energy storage in the nanothread bundle configuration (see additional discussions in Supplementary Note 3).

Compared with nanothreads, the theoretical predictions for CNT exhibit a relatively big deviation from the MD simulations. Here, the torsional energy density constant of 19.76 MJ kg^−1^ (with $\varepsilon _{\mathrm{t}} \,<\,0.06$) is adopted in the theoretical model. According to Fig. 5c, the theoretical predictions agree well with MD simulations at small strain (e.g. $\varepsilon _{\mathrm{t}}$ < 10%), but deviates from MD simulation results at the elastic limit. Such deviation originates from the nonlinearities induced by the twist-induced flattening, which also affect the bending, compression and tensile deformation of CNT. Despite that, torsion is also shown as the dominant deformation mode, but the contribution from the tensile deformation only increases slightly with the increase in filament number. It is noticed that the actual mechanical energy storage density never approaches the maximum magnitude (when all deformation modes meet their elastic limit) for both nanothreads and CNTs. For instance, the maximum gravimetric energy storage density is ~3.65 MJ kg^−1^ for nanothread-A bundles (from Table 1), which is significantly higher than that obtained from MD simulations (1.19 MJ kg^−1^ bundle-3) or theoretical predictions (0.99 MJ kg^−1^). Moreover, the gravimetric energy density decreases with the filament number. Such observations are due to the fact that different deformation modes are coupled in the bundle, and the energy storage limit is controlled by their failure mechanisms, which varies from bundle to bundle.

### Optimum mechanical energy storage in nanothread bundle

**Fig. 6:**
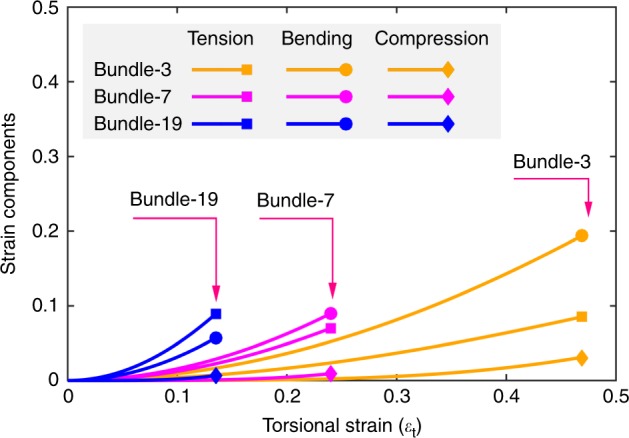
Different deformation strain components. The theoretical predictions of the strain components as a function of the torsional strain for nanothread-A bundles.

The above analyses suggest that the energy stored in a twisted nanothread bundle is likely to be well below the maximum value because it is assumed that all elastic limits are achieved. Under torsion, the outermost filaments experience the most severe tensile and bending deformation and all nanothread filaments experience the same amount of torsion. Assuming a same compressive strain between each pair of nanothreads, we can assess the strain status for the outermost nanothreads through the theoretical model (focusing on the small strain range). Take the nanothread-A bundle as an example, it is surprisingly that the bending strain grows faster than the tensile strain for the bundle with $n\, <\, 7$ (Fig. 6). Increasing the filament number further leads to a much larger coil radius for the outermost filaments, which results in a more rapid increase in the tensile strain (as seen in bundle-19). In comparison, the compressive strain exhibits a gradual increase for all nanothread-A bundles. Consistent results are observed for nanothread-C and CNT bundles (see Supplementary Fig. 8).

Based on the relationships between different deformation modes (Eq. 1), the ideal failure mode of nanothread bundles can be qualitatively assessed by examining which strain component reach its elastic limit first. It is found that the fracture of thinner bundles (*n* < 7) is limited by bending for nanothread-A, but by torsion for nanothread-C. For larger bundles, the elastic regimes of both nanothread-A and -C bundles are limited by tensile strain. CNT bundle is found to share similar fracture mechanisms as nanothread-A bundles (see Supplementary Fig. 9). The results above indicate that the energy stored in a twisted nanothread bundle is dominated initially by torsion, and the stored tensile energy rapidly dominates as the filament number increases. Although the bending strain shows a faster increase than the tensile strain for bundles with small filament number (*n* ≤ 7, see Fig. 6), the relatively small bending constant ($k_{\mathrm{b}}$) results in minor contribution to the total mechanical energy storage (Fig. 5). Recall in Table 1 the maximum energy storage for an individual nanothread under pure tension is much higher compared with other deformation modes (~2.05 and 0.91 MJ kg^−1^ for nanothread-A and -C, respectively). Thus, a straightforward way to maximize the energy storage capacity of nanothread bundle is to utilize the full tensile potential of each constituent nanothread filaments. It is noted that experimental studies have shown that the carbon nanothreads can be partially saturated^41^, which could increase their elastic limit^42^. This provides the possibility of using partially saturated nanothreads at the outermost layer of the bundle to enhance the mechanical performance of a twisted bundle. The torsional performance of the bundle structure could also be improved by adopting a multi-level hierarchical structure or introducing certain pre-strain to the outermost filaments.

**Fig. 7:**
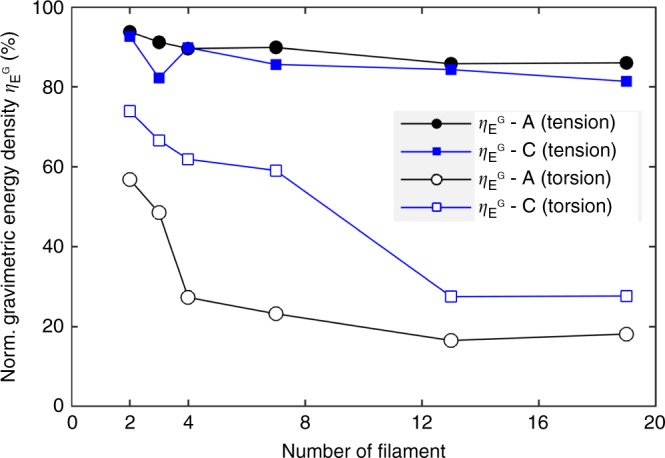
Normalized gravimetric energy density of the nanothread bundles from the simulation. Here A and C denote nanothread-A and nanothread-C, respectively.

To illustrate, we conducted another set of pure tensile MD simulation for nanothread-A and -C bundles with varying filament number. Comparing with torsion, a very high gravimetric energy density is observed for the tensile deformation of nanothread-A and -C bundles with 19 filaments (~1.76 and 0.81 MJ kg^−1^, respectively). From Fig. 7, the normalized gravimetric energy density ($\eta _{E^{\mathrm{G}}}$) of a bundle structure (either under tension or torsion) is lower than that of an individual structure under tension. Here, $\eta _{E^{\mathrm{G}}} = E_n^{\mathrm{G}}/E_{t1}^{\mathrm{G}}$ and $E_n^{\mathrm{G}}$ and $E_{\mathrm{t1}}^{\mathrm{G}}$ represent the gravimetric energy density of a bundle-*n* structure under torsion or tension, and the gravimetric energy density of the corresponding individual nanothread under tension, respectively. Under torsion, the gravimetric energy density experiences a remarkable reduction of ~40% for both nanothread-A ($\eta _{E^{\mathrm{G}}}$ decreases from ~57% to 18%) and nanothread-C ($\eta _{E^{\mathrm{G}}}$ decreases from ~73% to 28%) when *n* increases from 2 to 19. In comparison, the gravimetric energy density for both nanothread-A and -C under tension only shows a reduction of within 10%, for example, $\eta _{E^{\mathrm{G}}}$ decreases from 94% to 86%. The amount of reduction in the bundle structure under pure tension is regarded as originating from the change of inter-tubular interactions, which is different from the tension of an individual nanothread. We also extend the calculation for CNT bundles under pure tension and obtained similar results, that is, maintaining a high energy storage capacity with increasing number of filament (see Supplementary Fig. 10).

## Discussions

Generally, it is found that the gravimetric energy density of the nanothread bundle decreases with the number of filaments, and torsion and tension are the two main players for the mechanical energy storage. Specifically, tension dominates the energy storage for larger bundles. Though individual CNTs exhibits better mechanical properties than nanothreads, our results show that the nanothread bundle has a comparable mechanical energy storage capacity with CNT bundles under torsion. In particular, nanothread bundles exhibit their own advantages compared with CNT bundles in terms of mechanical energy storage. Firstly, CNT bundles experience structural instability at a very small strain, which does not occur in nanothread bundles. Secondly, the maximum elastic torsional deformation that the CNT can sustain is much smaller than that of the nanothread. Thirdly, the intrinsic structure of nanothread enables the possibility of activating the optimum mechanical energy storage capacity in the bundle structure through pure tension. This is due to the fact that the hydrogenated surface of nanothread bundle facilitates the establishment of inter-thread bonds. Experiments have already demonstrated the feasibility of functionalizing nanothreads with –NH_2_ groups^23^, which decorate the exterior of the nanothreads and acts as potential linkers for adjacent nanothreads. DFT calculations^43^ show the possibility of functionalizing with various functional groups (e.g. –CH_3_, –NH_2_ –OH, and –F), while retaining the mechanical properties of nanothreads.

It is noted that due to the low simulation temperature, the elastic limits of different deformation modes are likely overestimates of the behaviour of actual CNT and carbon nanothreads at room temperature, and consequently there is an overestimation of the energy storage capacity at room temperature. A future study is needed to justify the influence of temperature. Preliminary studies show that the mechanical properties of CNT(10,10)^44^ are less sensitive to temperature than that of the carbon nanothreads^28^. However, considering the large varieties of nanothreads, it is still an open question whether CNT bundles can outperform nanothread bundles at finite temperature, especially for the bundles constructed from thinner CNTs. Additionally, although the applied strain rate in this work is sufficiently small to allow confidence in the simulation results, it is still huge compared with that in experiments. Literature show that the elastic limit will increase slightly at higher strain rates, while the elastic modulus is normally insensitive to the strain rate^28,29^. As such, a slight reduction of energy storage capacity is expected under lower strain rates.

Overall, the gravimetric energy density of the nanothread bundle can reach 1.76 MJ kg^−1^ under pure tension, which is 4 to 5 orders higher than that of a steel spring (~0.14 kJ kg^−1^)^45^ and up to three times compared to Li-ion batteries (~0.43–0.79 MJ kg^−1^)^46^. An application for such pure tension scenario for bundle structures was proposed previously for CNT bundles^1^ based on an escapement mechanical system that is commonly seen in pendulum clocks^47^. With this mechanical system, the nanothread bundle can be connected by a right-handed and a left-handed screw. By turning the screw, the nanothread bundle will experience pure extension and thereby act as the mechanical energy storage system^1^. Considering the large variety of carbon nanothreads, nanothread derivatives (such as the carbon nitride nanothreads)^48^, and the huge variety of bundle configurations (with a mixture of threads of varying chirality), it is of great interest to carry out further experiments and theoretical works to explore the applications of carbon nanothread bundles for mechanical energy storage.

## Methods

### Sample selection and general settings

Theoretical calculations predict 50 kinds of carbon nanothreads, among which 15 have a relatively low energy and are classified into two classes, namely, chiral and achiral.^21^ For comparison purposes, two representative carbon nanothreads (one from each class) were selected in this work, including the achiral polymer I (135462), and the chiral nanothread 134562, which are denoted as nanothread-A and nanothread-C, respectively. The six integers (some underscored) represent the bonding topology in the structure, which is used as a standard nomenclature for nanothread^21^. It should be noted that the mechanical performance of the selected nanothreads is not expected as generalizable to other nanothreads, that is, the results in this work only intend to demonstrate the capability of the new carbon nanothreads for mechanical energy storage. A comprehensive understanding of the capacity of other carbon nanothreads requires future investigation.

In order to consider a larger sample size, large-scale MD simulations were adopted. The widely used AIREBO potential was employed to describe the C-C and C-H atomic interactions^49,50^. The potential includes short-range interactions, long-range vdW interactions (as described the Lennard–Jones term) and dihedral terms, which have been shown to well represent the binding energy and elastic properties of carbon materials. It accurately reproduces the elastic properties of carbon systems, such as the twisting of CNT^51^, and tension-twisting deformation of CNTs^52^, and has been reported to reasonably capture the vdW interactions in CNT bundles^53^. It is noted that the Lennard–Jones term is reported to be deficient in describing the *sp*^2^ interlayer interactions with changes in the interlayer registry^54^. However, this will not affect our results as this work focuses on DNTs and they are *sp*^3^ carbon structures. It is important to mention that AIREBO potential usually suffers from a non-physical high tensile stress under bond stretching due to the switching function^55^. A conventional way to overcome this is to extend the original cut-off value of 1.7 to ~1.9–2.0 Å^56,57^. In this work, a cut-off of 2.0 Å was adopted. It is expected that a higher elastic limit (before fracture or failure) would be obtained if a smaller cut-off was selected. A comprehensive discussion on the influence from different cut-off distances can be found in our previous work^29^. The real magnitudes of the mechanical properties are supposed to experience certain deviations from the MD results because of the nature of the empirical potential, which is usually fitted from experimental measurements or first-principle calculations when experimental data is absent.

To limit the influence of thermal fluctuations, a low temperature of 1 K was adopted for all simulations. This is also commonly applied in literature when investigating the mechanical properties of nanomaterials in order to remove the thermal influence. The literature shows that the temperature normally exerts small or negligible influence on the elastic modulus^28,58^, whereas the elastic limit will normally experience a nontrivial decrease when the temperature increases^28,36^. A small time step of 0.5 fs was used for all calculations with all MD simulations being performed using the software package LAMMPS^59^.

During the simulation, the commonly used virial stress was calculated, which is defined as^60^

3 $${\mathrm{{\Pi}}}^{\alpha \beta } = \frac{1}{{\varOmega }}\left\{ { - \mathop {\sum}\limits_i {m_iv_i^\alpha v_i^\beta + \frac{1}{2}\mathop {\sum}\limits_i {\mathop {\sum}\limits_{j \ne i} {F_{ij}^\alpha r_{ij}^\beta } } } } \right\}.$$

Here, *Ω* is the volume of the system as calculated from ${{\varOmega }} = {\sum} {\overline {\omega _i} }$ with $\overline {\omega _i}$ representing the effective volume of atom *i*; $m_i$ and $v_i$ are the mass and velocity of atom *i*; $F_{ij}$ and $r_{ij}$ are the force and distance between atoms *i* and *j*; and the indices *α* and *β* represent the Cartesian components. The atomic virial stress can be obtained by replacing the system volume with the effective volume of atom (i.e. $\overline {\omega _i}$) in Eq. (3). Considering the sophisticated stress status that the atoms experience in the deformed nanothread bundle, the atomic Von Mises stress was also calculated based on the atomic virial stress. The system volume of the nanothread is estimated by approximating an individual nanothread as a solid cylindrical beam with an effective radius of $r_0$. In this case, the equivalent cross-sectional area of a nanothread equals to *λV*_0_, with *V*_0_ as the reference atomic volume for carbon atom in bulk diamond (~5.536 Å^3^/atom)^61^ and *λ* as linear atom density (in the unit of atoms/Å)^21,62^. As such, the overall cross-sectional area of the nanothread bundle is approximated by *nλV*_0_, where *n* is the number of filaments. Adopting different approaches to calculate the volume of the nanothread bundle would lead to different absolute stress values, but not affect their relative magnitudes.

### Bundle model construction

Based on the equilibrium inter-tubular distance, different nanothread bundles are constructed with the filament number *n* varying from 2 to 19 (denoted as bundle*-n*). This filament number range is selected to enable full consideration of the close-packing morphology allowed by the triangular lattice without excessive computational costs. The CNT or carbon nanothread bundles has a close-packed configuration, that is, a triangular lattice, which has the lowest system energy and is being widely adopted for CNT bundles^63^. A series of relaxation simulations under isothermal–isobaric ensemble were performed to identify the inter-thread separation distances between two filaments. The inter-thread separation distance refers to the distance between the centres of the two adjacent parallel nanothreads. For instance, two carbon nanothread models with different initial distances in lateral and longitudinal directions, and different orientations (or relative angles) were examined. The inter-thread separation distance was ~16.91, 6.25 and 7.25 Å for CNT(10,10), nanothread-A and -C, respectively. The bundle structures were then constructed based on these inter-thread separation distance values following the triangular lattice.

### Torsional deformation

Individual nanothread samples or bundles were firstly optimized by the conjugate gradient minimization method and then equilibrated using the Nosé-Hoover thermostat^64,65^ for 1000 ps. Periodic boundary conditions were applied along the length direction during the relaxation process. Thereafter, a constant torsional load (i.e. 2*π*/12,000 rad/ps) was applied to both ends of the sample in opposite directions (equal to a period of 6000 ps along its axis) to continuously twist the sample. All torsion simulations were carried out by switching to non-periodic boundary conditions and one end of the sample was fully fixed with the other end free in the longitudinal or axial direction. A similar initial deformable length (~15 nm) was chosen for all examined structures.

### Tensile deformation

The nanothread samples or bundles were firstly minimized and equilibrized with periodic boundary conditions in the length direction. The periodic boundary conditions were then switched off and a constant velocity (0.005 Å/ps) was applied to one end of the sample with the other end being fixed. This introduces a constant tensile strain rate to the structure. The nanothread sample or structure has a deformable region with a length of ~15 nm. The applied constant velocity yields to a strain rate of 3 × 10^−7^ fs^−1^, which is very large if compared with in situ experiments. However, for the simulation with a time step of 0.5 fs, the sample was only elongated by 1.5 × 10^−5^% each simulation step, which is nearly a quasi-static deformation.

This work only focuses on the perfect bundle structure with same filaments, whereas tensile deformation in experiments may trigger slippage between filaments due to their different lengths or pre-existing structural defects. Assessing the slip phenomenon would require large bundle samples (e.g. hundreds of nm in diameter), which could consume huge computational resources for MD simulations. In this regard, the coarse-grain method previously being applied to probe the bundle behaviours of CNTs^66^, is a promising alternative approach, which will be discussed in our future work.

### Bending deformation

Two different approaches have been adopted to perform bending simulation. For nanothreads, an initial curvature was introduced to bend the sample. A short sample size ~4 nm was chosen in order to reach a relatively large bending curvature. Due to their relatively low bending stiffness, the curvature of the nanothread was maintained by bonding the sample to an idealized surface with the wall–atom interactions described by an LJ 9/3 potential, that is, $E = \xi [2\left( {\sigma /r} \right)^9/15 - \left( {\sigma /r} \right)^3]$. Here, *ξ* and *σ* were chosen as 0.65 eV and 2 Å, respectively^27^. Curvatures ranging from 0.006 to 0.14 Å^−1^ were considered for the nanothread. After energy minimization, the bending energy can be derived by comparing with the unbent nanothread adhered to the same idealized wall. No periodic boundary condition was applied during the simulation.

For CNT(10,10), an initial curvature was also introduced to bend the sample. With both ends of the sample being fixed, the bent CNT was optimized by the conjugate gradient minimization method. The bending energy is then derived by comparing with the unbent CNT. A sample size of 8 nm was adopted, with the curvature varying from 0.027 to 0.136 Å^−1^. For both nanothreads and CNT, the initial bent structures were built by assuming pure bending deformation, that is, the length of the central line of the sample was a constant.

### Radial compression

To probe radial compression, a close-packed infinite triangular lattice of the nanothread with sixfold symmetry was considered. The triangular lattice was constructed by first obtaining the equilibrium inter-tubular distance *d*_0_ through a series of relaxation simulations under isothermal–isobaric ensemble^25^, that is, tuning the model with different initial distances in lateral and longitudinal directions and orientations. For nanothread-A, the inter-thread separation distance is ~6.25 Å, which agrees well with previous simulation results and experimental measurements^20,48^. A larger *d*_0_ of ~7.25 Å was found for nanothread-C, which presumably is due to its helical morphology.

A series of five-stage simulation of the nanothread lattice was conducted under isothermal–isobaric ensemble (NPT, with constant atom number, constant pressure and constant temperature), that is, relaxation at pressure 0, compressing the lattice from zero pressure to pressure *P*, relaxation at pressure *P*, releasing the pressure from *P* to 0 and relaxation at zero pressure. During the simulation, a same lateral pressure (coupled in *x-* and *y*-axis) was applied simultaneously to the nanothread lattice. Each stage was performed for the same simulation time of 250 ps. A total of 38, 23 and 34 tests were conducted for nanothread-A, -C and CNT, respectively. Periodic boundary conditions were applied in all directions to the triangular lattice and the temperature was kept as 1 K. The nanothread triangular lattice was optimized by the conjugate gradient minimization method both prior and after the five-stage simulation.

## Supplementary information

Supplementary Information

Supplementary Movie 1

Supplementary Movie 2

Supplementary Movie 3

Supplementary Movie 4

Supplementary Movie 5

Description of Additional Supplementary Files

## Data Availability

The data that support the findings of this study are available from the corresponding authors on reasonable request.
