# Supplementary material for: High density mechanical energy storage with carbon nanothread bundle
Source: Nat Commun. 2020 Apr 20;11:1905. doi: 10.1038/s41467-020-15807-7 (PMC7171126; doi:10.1038/s41467-020-15807-7)
Supplement: Supplementary file 1 — Supplementary Information [file 41467_2020_15807_MOESM1_ESM.pdf]

Supplementary Information

Ultra-high Density Mechanical Energy Storage with Carbon Nanothread Bundle

Zhan *et al*.

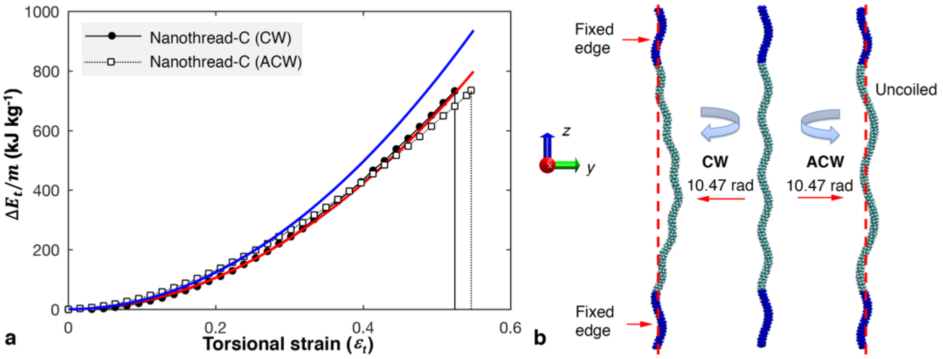

Supplementary Figure 1 | Torsional deformation of an individual nanothread-C under clockwise (CW) and anti-clockwise (ACW) torsion. (a) Torsional strain energy density as a function of dimensionless torsional strain. Red and blue solid lines are the fitting curves (based on Eq. 1) for CW and ACW torsion, respectively. Under anti-clockwise torsion, the MD data are best reproduced with a slightly higher torsional strain energy density constant around 3.10 MJ kg^{-1}, the elastic limit is similar as that under clockwise torsion; and (b) Atomic configurations of nanothread-C at a torsional angle of 10.47 rad. The two blue regions are the fixed edges. Clockwise torsion is found to further coil nanothread-C and introduces clear torsional buckling (with a clear deflection along its central axis). In comparison, the anti-clockwise torsion uncoils nanothread-C and induces a large pitch length (without obvious offset along the axis).

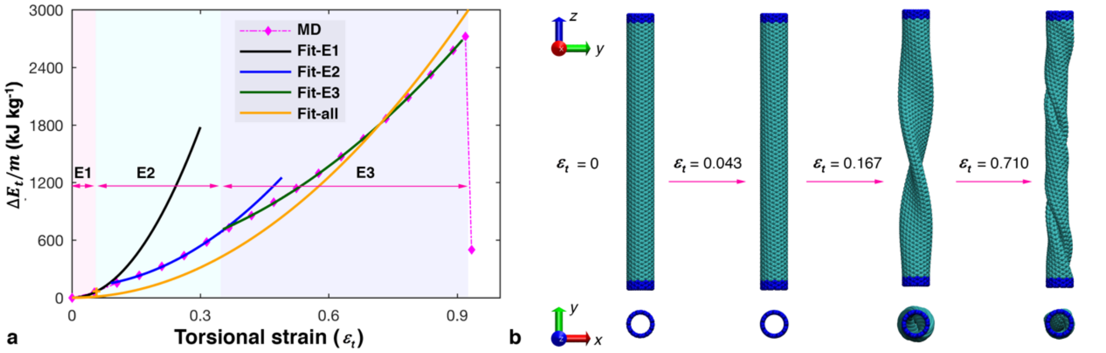

Supplementary Figure 2 | Torsional deformation of an individual CNT(10,10). (a) Torsional strain energy density as a function of dimensionless torsional strain. Solid lines are the fitting curves (based on Eq. 1) by considering different deformation stages, including stage I – initial torsion with circular cross-section, stage II – flattening, stage III – torsion with no further flattening (denoted as E1, E2, and E3, respectively). Fit-all represents the fitting within the whole elastic regime. The MD data in these three deformation stages are best reproduced by an energy density constant of about 19.76 MJ kg^{-1}, 4.74 MJ kg^{-1} and 2.78 MJ kg^{-1}, respectively; and (b) Atomic configurations of CNT at different deformation stages. Upper panels are the side views, and bottom panels are the end-on views. The two blue regions are the fixed edges. CNT keeps its circular cross-section at the first deformation stage (E1). With a very small dimensionless torsional strain (~0.06), CNT enters the second flattening deformation stage (E2) until a torsional strain of about 0.348. Thereafter, the CNT is fully flattened, and the structure is further deformed like a solid rod (the third deformation stage - E3).

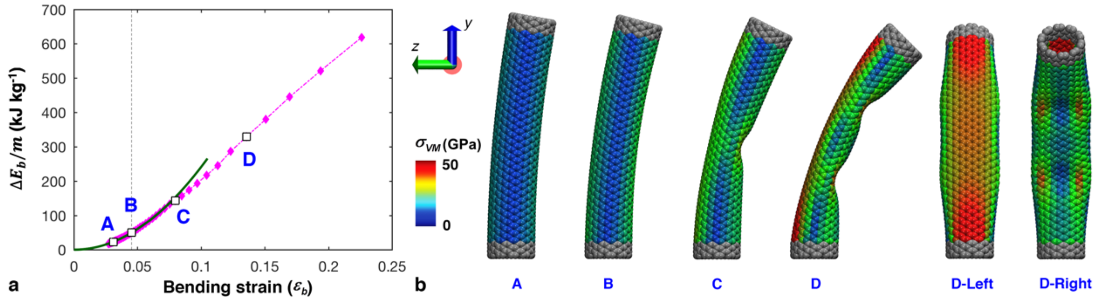

Supplementary Figure 3 | Bending deformation of an individual CNT(10,10). (a) Bending energy density as a function of bending strain. Solid lines are the fitting curves (based on Eq. 4); and (b) Atomic configurations of CNT at different strain values corresponding to the markers in a. D-left and D-right are the left and right views of CNT at the strain point D, respectively. Atoms are coloured according to Von Mises (VM) stress. It is seen that CNT experience buckling at a very small strain of around 0.05 under bending deformation. Clear stress concentration is observed when the structure is bent to a certain stage.

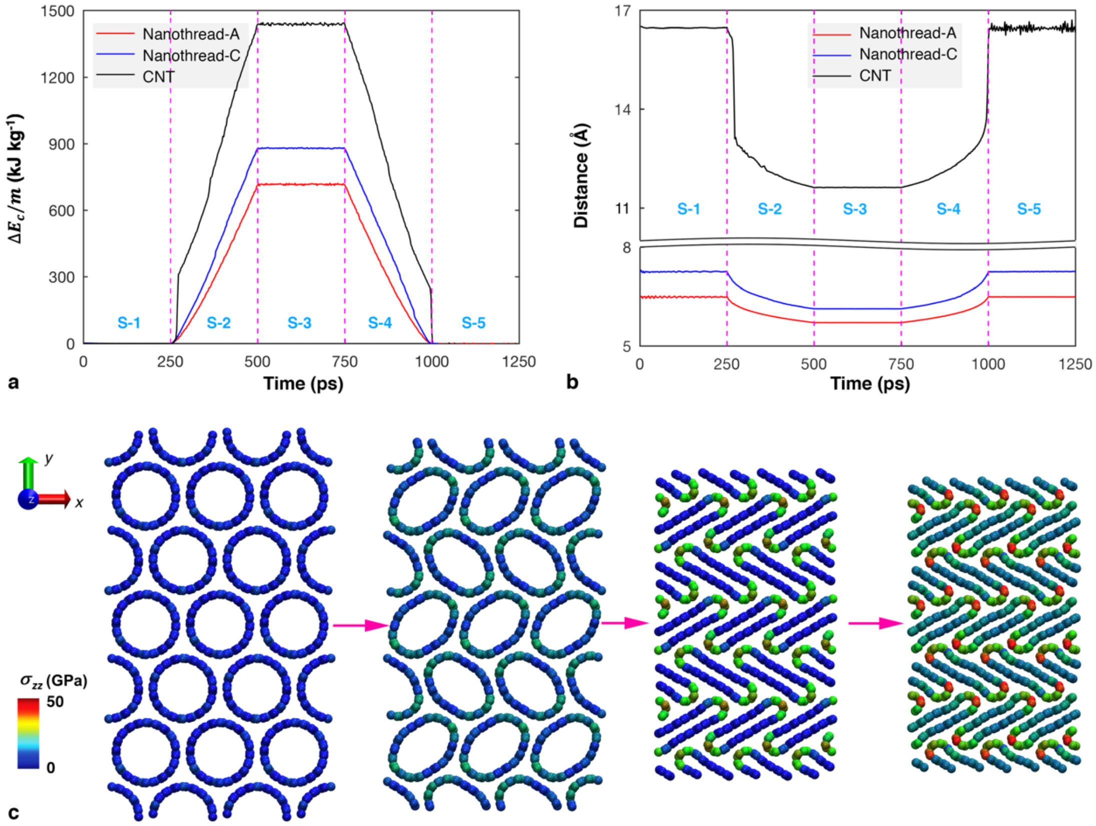

Supplementary Figure 4 | Compression of nanothread and CNT triangular lattice under radial compression. Five-stage simulation results at a pressure of 30 GPa for nanothread-A, -C and CNT: (a) Strain energy density as a function of time. Within the elastic regime, the strain energy increases/decreases when the pressure increases/decreases. Meanwhile, the separation distance decreases when the pressure increases from zero to *P*, and the nanothread structure can fully resume to its initial configuration. The energy profiles are highly symmetrical, indicating the reversible elastic deformation; (b) The separation distance *d* as a function of time, which experiences a reduction at pressure *P* (Stage III). Here the separation distance is tracked by estimating the distance between the central axes of two adjacent nanothreads. The sudden change of strain energy or inter-tubular distance during the pressure increasing (S-2) or decreasing (S-4) stage for CNT lattice is resulted from the flattening process; and (c) Representative atomic configurations illustrate the flattening of CNT under radial compression at stage 2 (S-2).

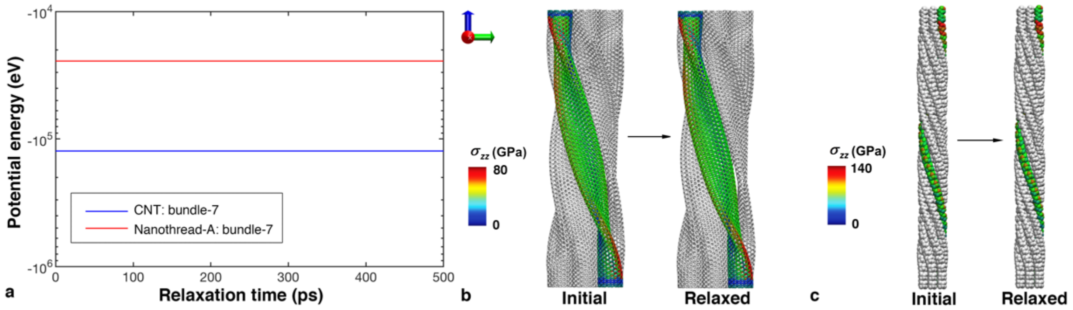

Supplementary Figure 5 | Comparison of nanothread and CNT bundle-7 under relaxation. (a) The potential energy of the bundle structure during the relaxation process. Comparisons of the atomic virial stress (in axial direction) between the initial structure and the structure after relaxation for: (b) the CNT bundle; and (c) the nanothread-A bundle.

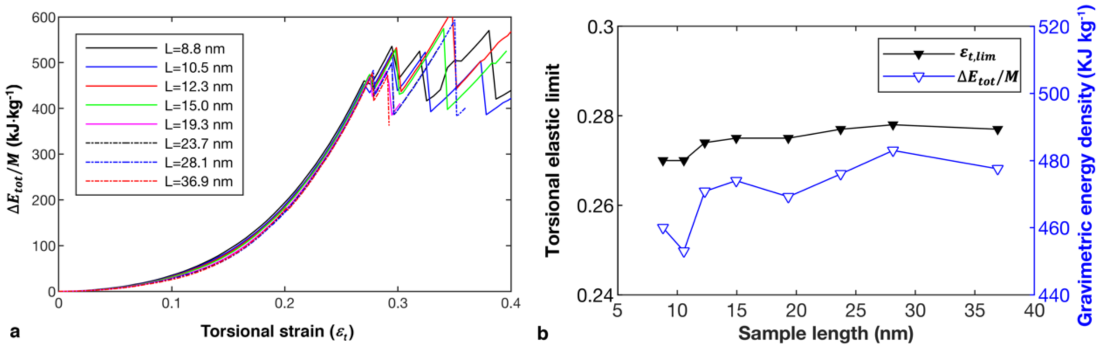

Supplementary Figure 6 | Twist deformation of nanothread-A bundle-7 with different sample lengths. The sample length increases from 8.8 nm to 36.9 nm. (a) The strain energy as a function of the dimensionless torsional strain. All profiles agree well with each other; and (b) A comparison of the torsional elastic limit and the gravimetric energy density as a function of sample length. The examined bundle structures exhibit a similar torsional elastic limit strain 0.275 ± 0.003 and gravimetric energy density 470 ± 9.8 kJ kg^{-1}, with no obvious length dependency for the examined sample length range.

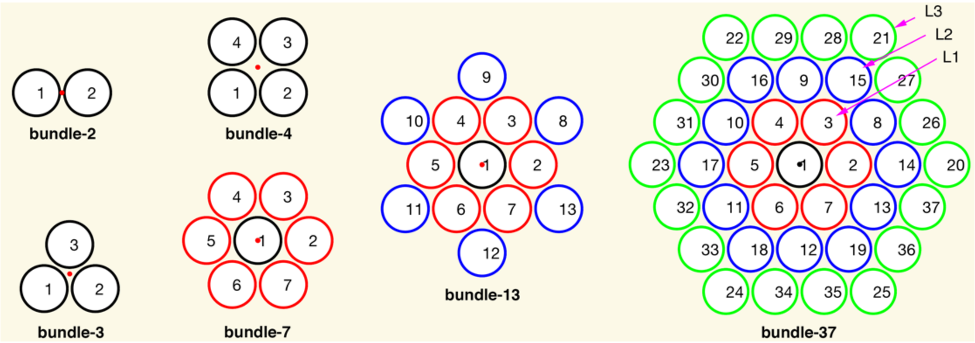

Supplementary Figure 7 | Schematic cross-sectional view of nanothread bundles with different number of filaments. Each circle represents one nanothread. Different colours correspond to different layers of nanothreads.

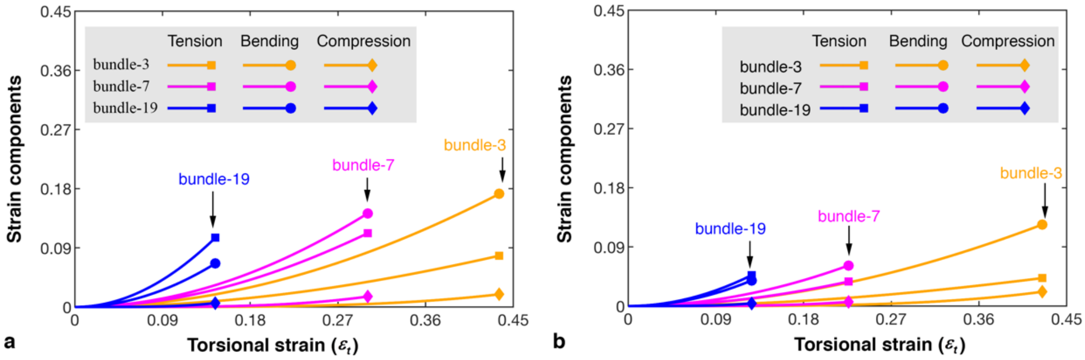

Supplementary Figure 8 | Different strain components as a function of the torsional strain. (a) Nanothread-C bundle; and (b) CNT bundle. Nanothread-C and CNT bundle share a similar changing tendency. Specifically, for bundle-3 and -7, the bending strain increases faster than the tensile strain. In comparison, the tensile strain takes the leading role in the bundle-19 structure.

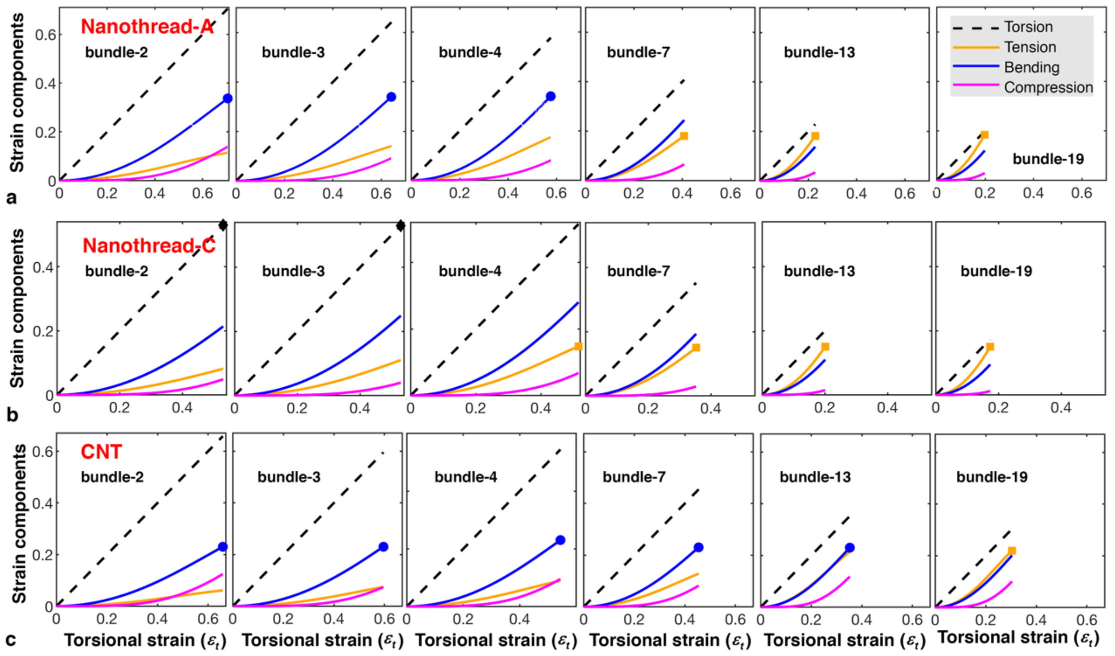

Supplementary Figure 9 | Theoretical failure mode of nanothread and CNT bundles. The tensile, bending and compressive strain as a function of the dimensionless torsional strain for: (a) Nanothread-A bundles, (b) Nanothread-C bundles, and (c) CNT bundles. The solid markers highlight the bundle reaches the corresponding elastic limit. As is seen, nanothread-A bundles experience bending-limited fracture when the filament number is smaller than 7. For larger nanothread-A bundles (n >= 7), the fracture is limited by tension. Although the fracture of larger nanothread-C bundle is also limited by tension, the failure of thinner bundle is found to be controlled by torsion. In comparison, CNT bundles share similar failure mechanisms as that of nanothread-A bundles. Note that the theoretical model is established based on linear elastic assumption, thus the failure mode results at large deformation are just for a qualitative assessment.

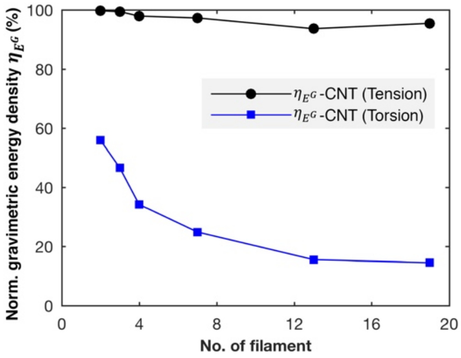

Supplementary Figure 10 | Comparison of the normalized gravimetric energy density of CNT bundle under torsion and pure tension. Here, the relative energy density is normalized based on the tensile elastic limit of an individual CNT. The gravimetric energy density decreases significantly under torsion when the filament number increases. Under pure tension scenario, the reduction is within 10%. Benefiting from the excellent tensile properties of individual CNTs, the CNT bundles can maintain a much higher gravimetric energy density (over 6.0 MJ kg^{-1}) compared with that of the carbon nanothread bundles. To note that current CNT bundles are normally fabricated through spinning or twisting to maintain a densely packed structure as a loose structure has low mechanical performance.[1] The low mechanical performance is majorly originated from low interfacial load transfer efficiency, which can be effectively enhanced by introducing certain percentage of initial torsion.[2] Researchers have tried to introduce inter-tubular covalent bonds between CNTs, which however results in detrimental effect on the mechanical properties of CNT bundles.[3]

Supplementary Note 1 | Fracture mechanisms of the bundle structure

For the bundle structure, the fracture usually initiates from the boundary region, which is expected due to the loading condition. Under torsion, the bonds at the interface needs to transfer the external load to the deformable region through bond torsion, which inevitably induce a local stress/strain concentration. In other words, the fracture behavior of the bundle is expected to be independent on the sample length, and the applied boundary condition would not affect our predictions. As such, the sample length is not expected to influence the torsional behavior of the bundle structure. To further affirmed such phenomenon, two sets of simulations have been performed, including: a relaxation simulation continued from the twisted bundle-7 structure (for both CNT and carbon nanothread) and the twist deformation simulation for nanothread-A bundle-7 with different sample length. For the relaxation simulation, the twist loads were removed, and the bundle was relaxed for 500 ps with the fixed boundaries. For nanothread-A, the atomic structure at the twist angle of 8.38 rad was selected. For CNT, the atomic structure at the twist angle of 2.09 rad was considered. As shown in **Supplementary Figure 5**, the potential energy of the bundle structure is very stable, suggesting no structural change during the relaxation process. By tracking the atomic configurations for CNT bundle and nanothread-A bundle, almost identical stress distribution patterns are observed before and after the relaxation. The stress concentration at the boundary regions are still existing after relaxation process.

Supplementary Note 2 | Theoretical description of the bundle deformation

The mechanical energy stored in the twisted nanothread bundle structure can be described by following the theoretical framework established by Tománek and co-authors for CNT,[4, 5] which is based on the Hooke's law with a consideration of linear elasticity. In detail, the total strain energy can be calculated from:

$$\Delta E/m = \frac{1}{n}\left[nk_t\varepsilon_t^2 + k_s\sum_{i=1}^{n}\varepsilon_{s,i}^2 + k_b\sum_{i=1}^{n}\varepsilon_{b,i}^2 + k_c\sum_{i<j}\varepsilon_{c,ij}^2\right] \tag{S1}$$

Here, $\varepsilon_t = \varphi D_0/l_0$; $\varepsilon_{s,i} = \sqrt{1+(\rho_i\varphi/l_0)^2} - 1$; $\varepsilon_b = D_0/R$, with $R_i = \rho_i[1 + (l_0/\varphi\rho_i)^2]$; $\varepsilon_{c,ij} = 1 - d_{ij}/d_0$; D_0 is the equivalent diameter of nanothread; and n is the filament number. Under the torsional deformation, each filament has a constant coil radius ρ_i, which is the equilibrium separation distance between the filament axis and the rope axis. The central nanothread only experiences torsion and compression. Assuming that the filaments form a regular polygon around the periphery of the bundle (see **Supplementary Figure 7**), for a radially compressed triangular lattice, the coil radius for each filament can be calculated from (by considering different layers L1, L2, and L3 in Supplementary Figure 7)

$$\rho_i = \begin{cases} \eta d_0\left(1-\varepsilon_{c,ij}\right) & 1 < i \le 7 \\ \sqrt{3}d_0\left(1-\varepsilon_{c,ij}\right) & 7 < i \le 13 \\ 2d_0\left(1-\varepsilon_{c,ij}\right) & 13 < i \le 19 \end{cases} \tag{S2}$$

Here, *i* corresponds to the ID of the filament as shown in Supplementary Figure 7 and $\eta = [2\sin(\pi/n_1)]^{-1}$. $n_1 = n$ for the bundle without central filament (e.g., bundle-3 in Figure S6), $n_1 = n - 1$ for the bundle with central filament (e.g., bundle-7, -13, or -19). Following Taylor series, the tensile strain

$\varepsilon_{s,i} = \sqrt{1+(\rho_i\varphi/l_0)^2} - 1 = \sqrt{1+\left(\varepsilon_{t,i}\rho_i/D_0\right)^2} - 1$, that is

$$\varepsilon_{s,i} \approx \frac{1}{2}\frac{\rho_i^2}{D_0^2}\varepsilon_{t,i}^2 = \begin{cases} \frac{\eta^2 d_0^2}{2D_0^2}\left(1-\varepsilon_{c,ij}\right)^2\varepsilon_{t,i}^2 & 1 < i \le 7 \\ \frac{3\eta^2 d_0^2}{2D_0^2}\left(1-\varepsilon_{c,ij}\right)^2\varepsilon_{t,i}^2 & 7 < i \le 13 \\ \frac{2\eta^2 d_0^2}{D_0^2}\left(1-\varepsilon_{c,ij}\right)^2\varepsilon_{t,i}^2 & 13 < i \le 19 \end{cases} \tag{S3}$$

The bending strain

$\varepsilon_{b,i} = D_0/\rho_i[1 + (l_0/\varphi\rho_i)^2] = \frac{\varepsilon_{t,i}^2\rho_i/D_0}{\left(\varepsilon_{t,i}\rho_i/D_0\right)^2+1}$, thus,

$$\varepsilon_{b,i} \approx \frac{\rho_i}{D_0}\varepsilon_{t,i}^2 = \begin{cases} \frac{\eta d_0}{D_0}\left(1-\varepsilon_{c,ij}\right)\varepsilon_{t,i}^2 & 1 < i \le 7 \\ \frac{\sqrt{3}d_0}{D_0}\left(1-\varepsilon_{c,ij}\right)\varepsilon_{t,i}^2 & 7 < i \le 13 \\ \frac{2d_0}{D_0}\left(1-\varepsilon_{c,ij}\right)\varepsilon_{t,i}^2 & 13 < i \le 19 \end{cases} \tag{S4}$$

Given the same (dimensionless) torsional strain ε_t and compression strain ε_c for all nanothread filaments in the bundle configuration, the tensile and bending deformation of the filament is the same within each layer for a same coil radius. Therefore, for the bundle with $1 < n \leq 7$, Eq. S1 can be approximated as:

$$\Delta E/m \approx \frac{1}{n}\left[nk_t\varepsilon_t^2 + n_1\frac{k_s}{4}\left(\frac{\eta d_0}{D_0}\right)^4(1-\varepsilon_c)^4\varepsilon_t^4 + n_1k_b\left(\frac{\eta d_0}{D_0}\right)^2(1-\varepsilon_c)^2\varepsilon_t^4 + n_ck_c\varepsilon_c^2\right] \tag{S5}$$

Here, n_c is the number of adjacent pair of nanothread filaments. Assuming a triangular lattice with central filament, the approximation procedure can be easily continued for bundles with higher filament number by considering the deformation separately in each layer. For example, for the bundle with $7 < n \leq 13$, the total strain energy equals to:

$$\frac{\Delta E}{m} \approx \frac{1}{n}\left[nk_t\varepsilon_t^2 + \frac{9n-57}{4}k_s\left(\frac{d_0}{D_0}\right)^4(1-\varepsilon_c)^4\varepsilon_t^4 + (3n-15)k_b\left(\frac{d_0}{D_0}\right)^2(1-\varepsilon_c)^2\varepsilon_t^4 + n_ck_c\varepsilon_c^2\right] \tag{S6}$$

Similarly, for the bundle with $13 < n \leq 19$, the total strain energy equals to:

$$\frac{\Delta E}{m} \approx \frac{1}{n}\left[nk_t\varepsilon_t^2 + (4n-37)k_s\left(\frac{d_0}{D_0}\right)^4(1-\varepsilon_c)^4\varepsilon_t^4 + (4n-28)k_b\left(\frac{d_0}{D_0}\right)^2(1-\varepsilon_c)^2\varepsilon_t^4 + n_ck_c\varepsilon_c^2\right] \tag{S7}$$

Evidently, the strain energy is a function of the torsional strain and compressive strain, i.e., $\Delta E_{tot}(\varepsilon_t, \varepsilon_c) = \Delta E/m$. For a given torsional strain ε_t, the optimum value of ε_c that minimizes the total strain energy can be obtained by solving $\partial E_{tot}(\varepsilon_t, \varepsilon_c)/\partial\varepsilon_c = 0$. That is, for $1 < n \leq 7$,

$$n_1k_s\left(\frac{\eta d_0}{z}\right)^4(1-\varepsilon_c)^3\varepsilon_t^4 + 2n_1k_b\left(\frac{\eta d_0}{D_0}\right)^2(1-\varepsilon_c)\varepsilon_t^4 - 2n_ck_c\varepsilon_c = 0 \tag{S8}$$

For $7 < n \leq 13$,

$$(9n-57)k_s\left(\frac{d_0}{D_0}\right)^4(1-\varepsilon_c)^3\varepsilon_t^4 + (6n-30)k_b\left(\frac{d_0}{D_0}\right)^2(1-\varepsilon_c)\varepsilon_t^4 - 2n_ck_c\varepsilon_c = 0 \tag{S9}$$

For $13 < n \leq 19$,

$$(16n-148)k_s\left(\frac{d_0}{D_0}\right)^4(1-\varepsilon_c)^3\varepsilon_t^4 + (8n-56)k_b\left(\frac{d_0}{D_0}\right)^2(1-\varepsilon_c)\varepsilon_t^4 - 2n_ck_c\varepsilon_c = 0 \tag{S10}$$

Supplementary Note 3 | Limitations of the theoretical model

The theoretical model is intended for a quantitative analysis of the strain energy storage with a small strain as it is established within the linear elastic regime. For large deformation, it can only provide a qualitative analysis. The inherent material nonlinearities at large strain and the geometrical nonlinearities that are out of a simple parabolic description are ignored in the theoretical model. A small deformation approximation is adopted when calculating the tensile and bending strains. There are no other or fitting of parameters in the theoretical model. Although the theoretical model is of limited value for quantitative prediction at large deformation, it is useful for rapid and qualitative assessment of the contributions to energy storage in the nanothread bundle. Additionally, though the theoretical model is established based on ultra-thin bundle structures, the relationships between different strain components will be the same for larger bundles in experiments. In other words, it can be predicted that for larger bundles, tensile deformation will be the dominant deformation mode when the bundle is under twist. We should highlight that the theoretical model has assumed an ideal bundle with uniform filaments. Experimentally, the bundle may contain different types of filaments, the filaments may differ in length, or some filaments may be defective. All these different bundles deserve a substantial research effort in the future, and the theoretical predictions may serve as the upper limit of their mechanical performance.

Supplementary References

1. Hill FA, Havel TF, Livermore C. Modeling mechanical energy storage in springs based on carbon nanotubes. *Nanotechnology* **20**, 255704 (2009).
2. Anike JC, Belay K, Abot JL. Effect of twist on the electromechanical properties of carbon nanotube yarns. *Carbon*, (2018).
3. O'Brien NP, McCarthy MA, Curtin WA. Improved inter-tube coupling in CNT bundles through carbon ion irradiation. *Carbon* **51**, 173-184 (2013).
4. Teich D, Fthenakis ZG, Seifert G, Tománek D. Nanomechanical Energy Storage in Twisted Nanotube Ropes. *Phys Rev Lett* **109**, 255501 (2012).
5. Fthenakis ZG, Zhu Z, Teich D, Seifert G, Tománek D. Limits of mechanical energy storage and structural changes in twisted carbon nanotube ropes. *Phys Rev B* **88**, 245402 (2013).
