# Supplementary material for: High density mechanical energy storage with carbon nanothread bundle
Source: Nat Commun. 2020 Apr 20;11:1905. doi: 10.1038/s41467-020-15807-7 (PMC7171126; doi:10.1038/s41467-020-15807-7)
Supplement: Supplementary file 7 — Description of Additional Supplementary Files [file 41467_2020_15807_MOESM7_ESM.pdf]

Supplementary Information

Ultra-high Density Mechanical Energy Storage with Carbon Nanothread Bundle

Zhan *et al.*

Supplementary Figures

- **Figure 1 |** Torsional deformation of an individual nanothread-C under clockwise (CW) and anti-clockwise (ACW) torsion.
- **Figure 2 |** Torsional deformation of an individual CNT(10,10).
- **Figure 3 |** Bending deformation of an individual CNT(10,10).
- **Figure 4 |** Compression of nanothread and CNT triangular lattice under radial compression.
- **Figure 5 |** Comparison of nanothread and CNT bundle-7 under relaxation.
- **Figure 6 |** Twist deformation of nanothread-A bundle-7 with different sample lengths.
- **Figure 7 |** Schematic cross-sectional view of nanothread bundles with different number of filaments.
- **Figure 8 |** Different strain components as a function of the torsional strain.
- **Figure 9 |** Theoretical failure mode of nanothread and CNT bundles.
- **Figure 10 |** Comparison of the normalized gravimetric energy density of CNT bundle under torsion and pure tension.

Supplementary Note

- **Note 1 |** Fracture mechanisms of the bundle structure
- **Note 2 |** Theoretical description of the bundle deformation
- **Note 3 |** Limitations of the theoretical model

Supplementary Videos

- **Video 1 |** Compression and relaxation of nanothread-A triangular lattice: only the central filament is shown, and atoms are coloured according to the axial virial atomic stress in the range of 0 to 100 GPa.
- **Video 2 |** Compression and relaxation of nanothread-C triangular lattice: only the central filament is shown, and atoms are coloured according to the axial virial atomic stress in the range of 0 to 110 GPa.
- **Video 3 |** Torsional deformation of nanothread-A bundle-19: atoms are coloured according to the axial virial atomic stress in the range of 0 to 80 GPa.
- **Video 4 |** Torsional deformation of nanothread-C bundle-19: atoms are coloured according to the axial virial atomic stress in the range of 0 to 80 GPa.
- **Video 5 |** Torsional deformation of CNT bundle-19: atoms are coloured according to the Von Mises stress in the range of 0 to 60 GPa.
